# Data workflows and visualization in support of surveillance practice

**DOI:** 10.3389/fvets.2023.1129863

**Published:** 2023-02-09

**Authors:** Wiktor Gustafsson, Fernanda C. Dórea, Stefan Widgren, Jenny Frössling, Gema Vidal, Hyeyoung Kim, Wonhee Cha, Arianna Comin, Ivana Rodriguez Ewerlöf, Thomas Rosendal

**Affiliations:** Department of Disease Control and Epidemiology, National Veterinary Institute, Uppsala, Sweden

**Keywords:** animal health, epidemiology, data-driven, dashboards, digitalization, automation, reproducibility

## Abstract

The Swedish National Veterinary Institute (SVA) is working on implementing reusable and adaptable workflows for epidemiological analysis and dynamic report generation to improve disease surveillance. Important components of this work include: data access, development environment, computational resources and cloud-based management. The development environment relies on Git for code collaboration and version control and the R language for statistical computing and data visualization. The computational resources include both local and cloud-based systems, with automatic workflows managed in the cloud. The workflows are designed to be flexible and adaptable to changing data sources and stakeholder demands, with the ultimate goal to create a robust infrastructure for the delivery of actionable epidemiological information.

## Introduction

Prevention, detection and control of infectious diseases to safeguard animal health rely on the timely collection of evidence, and delivery of this evidence in formats that can be used for effective decision-making. In response to the growing availability of digital data sources which can be used to produce health intelligence, epidemiology progressively incorporates methods of big data analytics, developing digitalization workflows to convert a great variety of data into actionable epidemiological information.

To be useful in disease surveillance, however, these workflows need to be implemented continuously and remain true and relevant as not only data evolves, but also demands from stakeholders and knowledge itself. Hence, reusable and adaptable workflows for epidemiological analysis and dynamic report generation are required.

At the Swedish National Veterinary Institute (SVA), we strive toward automation to better fulfill our surveillance and knowledge communication responsibilities. We have a vision to move away from multiple parallel or manual workflows toward a common set of reusable tools, backed up by a robust infrastructure of cloud-based as well as local systems. The epidemiology team at SVA brings together different areas of knowledge, including epidemiology, software development and statistical modeling. We work closely with the community of internal and external users to ensure that the delivered tools and information serve the intended purpose.

Making tasks reproducible in a collaborative environment is challenging. Collaboration is a prerequisite, both to reduce person-dependence and because several areas of expertise are required to perform complex data analysis. Parallel development of analytical workflows often results in multiple solutions to similar problems. To avoid that, we aim to create common and shareable building blocks that can be reused in various applications. Here we describe a collaborative workflow centered on a joint development environment, common tools and a goal to reduce effort and improve reliability of results.

## Workflow components

**Figure 1:**
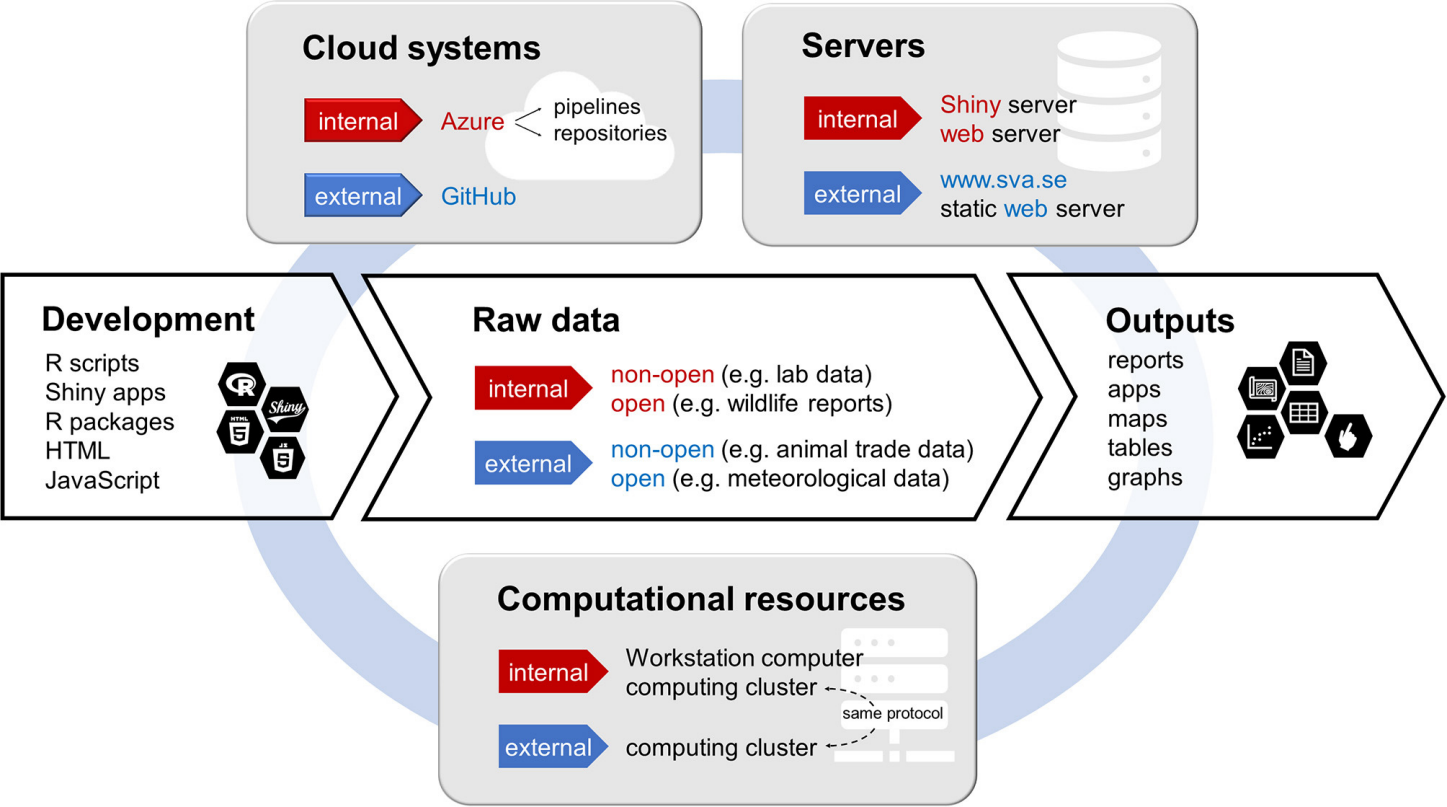
Schematic representation of the components of the described collaborative environment for data analyses. The main workflow from development to output (white background) operates through several supporting infrastructures (gray background). The supporting systems marked as “internal” are accessible by SVA only while “external” systems are publicly available. For the raw data, the distinction between internal and external instead refers to the data origin and ownership.

The components of our analysis and visualization workflows can be divided into four categories: (1) data access, (2) development environment, (3) servers and computational resources, and (4) cloud-based management. In general, development, version control and execution happen locally while the cloud environment is used for storage and administration of automatic workflows. See Figure 1 for an overview of the components and their interconnections.

### Data access

Examinations and tests from our laboratories are entered into a laboratory information management system. Information includes analyses performed, test results, animal species and geographical origin of the samples (coordinates and/or administrative region). The data are accessed through “data dumps” from a system of curated reports which are fed by database queries.

Additionally, we use several open data sources which are accessed directly through application programming interfaces (APIs). One such source is the SVA “Rapportera Vilt” system^1^ where anyone in Sweden can report findings of dead, sick or injured wild animals.

### Development environment

Development of scripts and workflows is done locally on personal computers. Code collaboration and backup is enabled through Git, a distributed revision control system (1). Each user makes changes (*commits*) on a local copy of the code in question. When ready, the local changes are then published (*pushed*) to a remote “origin” repository from which other users can retrieve (*pull*) the new revisions. This allows several colleagues to work on the same project in parallel while maintaining a common version history and avoiding the risk of undoing each other's work.

The main programming environment that we use is the R language for statistical computing (2) due to its familiarity in the group and wide support within the fields of data science, statistics and data visualization. We have developed R packages for specific purposes, e.g., data cleaning, report production and disease spread simulation, some of which are published publicly on GitHub^2^ or on the CRAN archive^3^ (see (3) as an example of such a package). We use static templates written in HTML and JavaScript to produce web content including maps, graphs and tables, which can be populated with up-to-date cleaned data on demand. We also build web applications using the R Shiny package (4), which enables the development of powerful and user-friendly web-based tools in R without the need for extensive skills in web development. An R Shiny application can be extended with custom HTML, CSS and JavaScript, which makes the environment especially flexible.

### Servers and computational resources

Personal computers are used for development and programming but are not ideal for execution of more computationally intensive tasks or for running automated analysis workflows. To solve this, we have two additional computational resources which are accessible for the whole department.

The first is a workstation computer running Microsoft Windows, which users can access through a remote desktop connection. This allows for the flexibility of working from a personal computer as well as the familiarity of the Windows environment while providing the user with additional processing power. The workstation is connected to all systems which any personal computer on the network can access, including network disks and internal web servers. Therefore, it is also used to run automatic workflows including the daily update of our web content on the current disease situation which requires access to internal data sources.

The second resource available is a cluster of computers running Linux, which are accessed by remote connection to a central node using the SSH protocol (5). This cluster is equipped with the SLURM Workload Manager (6), a scalable cluster management system in which the user can add jobs (scripts with instructions) to a queue. Once they are available, the requested resources will be allocated and the job is executed, without requiring the user to be actively logged in. Intensive jobs that do not include sensitive data can also be sent for execution in a similar system available at national level (Swedish National Infrastructure for Computing, SNIC) (7).

A deployment of ShinyProxy (8) on an internal server is used to host applications developed in the R Shiny framework. Each application is developed in the R package structure and subsequently built into an image which runs the application *via* the Docker (9) runtime. An image contains the application code itself and all its specific dependencies. Built application images are stored in a container registry which allows ShinyProxy to pull the latest version during the development phase and images to be tested locally for debugging.

### Cloud-based management

We use the Microsoft Azure DevOps cloud environment (10) for management of code and analytical workflows. In Azure, work is divided into projects which can be managed independently of each other. Each project contains one or several Git repositories as well as *pipelines* which are sets of instructions used to execute procedures in Azure (see examples of such procedures in the “Practical examples” Section).

The Azure DevOps projects are home to the remote origins of most of our Git repositories, for storage of scripts, R packages and content templates. For data sovereignty reasons, we do not store data on the Azure platform. The Git repositories may be directly connected to pipelines, of which there are two types: *build* and *release*. Build pipelines trigger automatically, either when new changes are pushed to the corresponding repository or on a regular time schedule and produce an output called an *artifact*. Release pipelines consume these artifacts and publish their contents. The publication location is typically a static web server that can be linked to from SVA's external website. The Azure system also has a container registry for the storage of containerized application images, which contain all dependencies for a specific application and can be downloaded and run locally (e.g., on the internal ShinyProxy server).

While pipelines and code are stored and managed in the cloud, some of the pipeline processes must be run locally to access internal data sources. A *pipeline agent* has been configured to run on the workstation computer with access to the required resources. Whenever a build pipeline configured to run on this agent is triggered, the Azure system sends the pipeline instructions and code to the local workstation for execution. The resulting artifact is then sent back to the cloud for publication. In this way, we can keep the flexibility of cloud-based management while maintaining data sovereignty.

## Practical examples

Below, we have highlighted several projects and activities where this environment of tools and methods has been employed in practice.

### Daily surveillance and disease situation summaries

The latest surveillance results generated at the laboratories are published daily to SVA's external website.^4^ The data are visualized in interactive graphs, maps and tables, and cover a range of disease agents of interest—including but not limited to chronic wasting disease in cervids, avian influenza in wild birds and African swine fever in wild boar. This workflow is managed by an R package designed specifically for this purpose, which contains HTML, JavaScript and CSS content template files as well as tools to analyze data and deploy the final content. The R package is hosted and code is executed in the Azure cloud environment. Every morning, a time-scheduled build pipeline is triggered in Azure. The pipeline instructs the department workstation to pull the latest changes from the git repository and execute the deploy scripts. Data are fetched from our internal systems, cleaned, summarized and combined with the appropriate templates to produce HTML files. These files are published back to Azure as an artifact, triggering a release pipeline which publishes them to our static web server. The latest update can be viewed on the SVA website as soon as this workflow has finished.

The disease situation webpages keep disease control experts, animal owners and the public informed about the current Swedish animal disease situation. This timely communication is important for the public who otherwise would only have access to periodic data summaries in agency reports. Continuous updates of data have also facilitated the early detection of new trends and outbreaks, which contributes to a robust surveillance.

### Annual surveillance report

*Surveillance of Infectious Diseases in Animals and Humans in Sweden*^5^ is an annual report describing the Swedish surveillance activities during the previous year, covering important animal pathogens as well as select zoonotic agents in a One Health context. It is published by SVA in collaboration with the Swedish Board of Agriculture, the Public Health Agency and the Swedish Food Agency (11).

The report is divided into chapters, one per disease agent or topic covered. The responsible authors write their chapters in word processing software and provide data for figures in spreadsheets in a cloud environment. These documents and spreadsheets are then converted to the LaTeX (12) document preparation system using a fully open-source “report engine” built as an R package that depends on the Pandoc (13) document conversion software. An Azure DevOps pipeline stitches together the chapters in a LaTeX report template and produces a PDF document, which is published to our external web server.

This system facilitates the work of the authors, as they work in a familiar collaboration-friendly environment decoupled from the final typesetting of the report. The authors, from different agencies with access to different data, can work closely to create a synthesis of the annual surveillance results without sharing raw data with each other. Additionally, everyone is involved in the design process since the latest PDF version is always available for them to view online.

### SvarmIT—Interactive tool for antimicrobial resistance surveillance

SvarmIT is a tool developed to visualize up-to-date antimicrobial resistance (AMR) data from 2010 and onward for different sample types, bacterial species, antibiotics and animal species. The trends are shown in relative frequency of resistant isolates among the total tested in a year, which can be exported as a graph or table for further analysis. A daily workflow was set up to clean, analyze and aggregate susceptibility testing data using R, and attach it to an HTML/JavaScript template which is published to our external website^6^. Additionally, SvarmIT sends a notification to the laboratory personnel when there are new samples that need further investigation based on the initial phenotypic test results.

Continuous monitoring is a fundamental part of the work to stop and prevent the spread of AMR. Every year, SVA examines ~12,000 samples from animals for the presence of antibiotic-resistant bacteria and results. SvarmIT is designed to meet the FAIR^7^ principles; it is accessible to the public and facilitates the delivery and communication of daily AMR surveillance results. Previously, stakeholders would only have access to the compiled summaries published in the annual Swedres-Svarm report (14). SvarmIT also contributes to a better collaboration and a robust and more timely AMR processing workflow within SVA, thereby enhancing the quality of the surveillance.

### Anthrax dashboard

The Anthrax dashboard is an interactive graphical tool to visualize historical outbreaks that occurred in Sweden from 1916 to 2016 based on information from Swedish archives (15), along with relevant weather data (16). The application was built using JavaScript, particularly two major libraries: Leaflet (17) and D3 (18). The workflow to clean, analyze and convert the historical data to JSON format was written as an R package. The dashboard is hosted on SVA's internal web server.

Following an outbreak of anthrax, bacterial spores can remain dormant in the soil and can cause new infections in susceptible grazing animals for decades (19). For decision-makers, the dashboard is a useful tool to quickly evaluate the risk of anthrax in a specific area based on whether an outbreak has previously occurred nearby.

### *Salmonella* portal

The *Salmonella* portal is a dashboard where all surveillance activities for the disease agent are collected. It presents the historical and present surveillance results of *Salmonella* in Swedish animals, including production animals, wildlife and domestic cats. The dashboard is developed in R Shiny and hosted on the internal ShinyProxy server. *Salmonella* has been selected as a pilot case and future development is planned for several other disease agents of importance. The dashboard is therefore developed with reusability in mind, with the aim to create a main template layout. It is currently designed for internal use but will eventually be public.

The daily surveillance results are visualized in maps, graphs and tables, allowing users to browse the data and potentially identify spatiotemporal patterns. The annual surveillance report (11) summaries are also presented here, along with general information about the agent and the existing surveillance programs. This resource provides decision-makers and the public with an up-to-date comprehensive view of *Salmonella* surveillance in Sweden, leading to a better understanding of the disease over time and across several species.

### Applications developed in collaboration with external stakeholders

In addition to the examples mentioned above, SVA also works on the development of several applications for users in the industry, such as farmers and veterinary advisors. The development is iterative, with regular interaction with users in the form of focus groups, surveys, workshops, prototype evaluations and meetings. Hence, user requirements and tool functionality also evolve during the development phase, with scalability and flexibility being key requirements.

The applications use data from SVA's laboratory systems as well as from external sources. Data used in these applications include test requisitions, test results, health and reproductive events, and productive performance of the animals. In some applications, the sensitive nature of the data included requires different user profiles with different levels of access to the information. Applications that are used by in-house advisors are hosted in our internal ShinyProxy server. In the future, development plans include the search for hosting solutions that will allow access by users outside the institute. These development projects allow both the data owners and SVA to demonstrate how the additional value of animal health data can be captured through interactive applications.

## Discussion

Data analysis processes to transform data into information that can support animal disease surveillance have often been discussed in literature, but implementation of these in automated workflows that can be employed continuously or on-demand presents several challenges. Our experiences described here show that collaboration is required to have robust and sustainable workflows and to handle the complexity in data management and analysis in the best way.

Establishing reusable functionality is important for improving efficiency and quality control. However, it has been challenging to identify core functions that should be built as reusable components in a code library that is shared between projects. An optimal solution can never be achieved but the group must continuously work toward improvement. Currently, close collaboration with frequent communication has been used to update coworkers on what core functions are implemented for reuse. An ongoing challenge is to identify if these core functions are actively being used in new projects. Code review is not a tradition in epidemiology, and as it costs substantial time it is usually not prioritized. However, our goal to use the R package structure for collecting our work allows us to take advantage of R's available tools for code testing to guide improvements and maintain quality control.

The use of centralized cloud platforms for code storage and execution of processes has helped enable collaborative development and reduce the person-dependence of recurring tasks. It has allowed the transfer of responsibilities more efficiently during vacation periods or during periods of high workloads such as disease outbreaks. However, the use and maintenance of these systems rely on the availability of human resources, and prior planning is necessary to ensure that personnel have the appropriate skills to complete the required tasks or debug problems that may occur. Proficiency in programming and tools such as version control is not normally expected of veterinary epidemiologists and has required substantial training.

The current landscape for practical work in epidemiology is changing rapidly with the introduction of new tools and strategies primarily adapted from methods used in software development. This requires education to establish these new skills and close collaboration within a team. At SVA, we have advanced our working strategies over recent years but acknowledge that continuous effort is required to maintain and continue to improve how we work. These modern approaches support the timely and accurate reporting of surveillance results to the public as well as forming a sound foundation for expert evaluation of trends or other changes in the Swedish animal disease situation.

## Data availability statement

The original contributions presented in the study are included in the article/supplementary material, further inquiries can be directed to the corresponding author.

## Author contributions

WG is the main responsible for the implementation and maintenance of pipelines in Azure DevOps. JF is the group leader. FD, WG, and TR prepared a first draft of this manuscript, which was reviewed, and amended and approved by all other authors. All authors participate actively in the development, maintenance and use of data workflows at the Department of Disease Control and Epidemiology.

## Footnotes

^1^ https://rapporteravilt.sva.se/

^2^ https://github.com/sva-se/

^3^ https://cran.r-project.org/

^4^ https://www.sva.se/amnesomraden/smittlage/

^5^ https://www.sva.se/amnesomraden/smittlage/sjukdomsrapporter-om-sva-s-overvakning/

^6^ https://www.sva.se/en/our-topics/antibiotics/svarm-resistance-monitoring/svarmit-interactive-resistance-monitoring-tool/

^7^ https://www.go-fair.org/fair-principles/
